# Sustained response in early responders to safinamide in patients with Parkinson's disease and motor fluctuations: A *post hoc* analysis of the SETTLE study

**DOI:** 10.3389/fneur.2023.1147008

**Published:** 2023-03-27

**Authors:** Roongroj Bhidayasiri, Michinori Koebis, Takanori Kamei, Takayuki Ishida, Ippei Suzuki, Jin Whan Cho, Shey-Lin Wu

**Affiliations:** ^1^Chulalongkorn Centre of Excellence for Parkinson's Disease and Related Disorders, Department of Medicine, Faculty of Medicine, Chulalongkorn University and King Chulalongkorn Memorial Hospital, Thai Red Cross Society, Bangkok, Thailand; ^2^The Academy of Science, The Royal Society of Thailand, Bangkok, Thailand; ^3^Medical Headquarters, Eisai Co., Ltd., Tokyo, Japan; ^4^Clinical Evidence Generation Fulfillment, Deep Human Biology Learning, Eisai Co., Ltd., Tokyo, Japan; ^5^Department of Neurology, Sungkyunkwan University School of Medicine, Seoul, Republic of Korea; ^6^Neuroscience Center, Samsung Medical Center, Seoul, Republic of Korea; ^7^Department of Neurology, Changhua Christian Hospital, Changhua, Taiwan

**Keywords:** safinamide, Parkinson's disease, levodopa, monoamine oxidase B inhibitor, motor fluctuation, *post hoc* analysis, treatment response

## Abstract

Safinamide is a selective, reversible, monoamine oxidase B inhibitor for the treatment of patients with Parkinson's disease (PD) and motor fluctuations. This was a *post hoc* analysis of the SETTLE study, in which patients with PD and motor fluctuations were randomly assigned to 24-week treatment with safinamide (50 mg/day for 2 weeks, increased to 100 mg/day if tolerated) or placebo. In the present analysis, responders were defined according to their treatment responses at Week 2 and Week 24 based on changes in ON-time without troublesome dyskinesia from baseline with cutoffs of 1 hour. It was found that 81% (103/127) of the responders at Week 2 maintained the response through Week 24 in the safinamide group. Other outcomes did not necessarily coincide with the ON-time response; however, “Early” responders who showed a treatment response at both Week 2 and Week 24 had substantial improvements from baseline in OFF-time, UPDRS Part II and III scores, and PDQ-39 summary index scores through Week 24. The safinamide group had a higher proportion of early responders than the placebo group (39% vs 20%, *p* < 0.0001). At baseline, early responders in the safinamide group had significantly higher UPDRS Part II and III scores, shorter ON-time, and longer OFF-time than the other responder populations. In conclusion, the results of the present *post hoc* analysis suggest that patients with a short ON-time, severe motor symptoms, and highly compromised activities of daily living can benefit from safinamide early in treatment and over the long term.

## Introduction

Parkinson's disease (PD) is a neurodegenerative disorder characterized by the loss of dopaminergic neurons in the substantia nigra of the midbrain. Dopamine replacement therapy with levodopa is the gold standard pharmacotherapy for PD; however, with pulsatile dopaminergic stimulation by levodopa and disease progression, motor fluctuation or wearing-off develops (1).

The onset of wearing-off often requires additional adjunct treatment to levodopa, such as dopamine agonists, monoamine oxidase B inhibitors, or catechol-O-amine transferase inhibitors. Although these drugs have been shown in clinical studies to be superior to placebo in reducing wearing-off, not all subjects achieve clinically significant changes (2, 3). In clinical practice, the treatment of PD is individualized, depending on various factors, including patients' demographic characteristics, symptoms manifested and their severity, concomitant drugs, and/or comorbidities (4, 5). Several subgroup analyses have been conducted according to these factors, showing robustness of treatment efficacy and safety [e.g., (6–10)]. However, few have successfully characterized responders to treatment (10, 11) by methods other than the pharmacogenomic approach (12–14).

Safinamide is a monoamine oxidase B inhibitor that is unique for its reversible monoamine oxidase B inhibition and voltage-dependent sodium channel blockade effect (15), and its efficacy in reducing wearing-off has been demonstrated by three Phase III trials and a Phase II/III trial (16–19). In these trials, 57.7% to 64.3% of subjects showed a clinical response to 24-week treatment with safinamide 100 mg/day, as defined either by a 1-h or greater increase in ON-time without troublesome dyskinesia or by Clinical Global Impression**—**Change. The responder rate was significantly greater than with placebo (25.7 vs. 48.1–61.7%) (18). A meta-analysis of the trials demonstrated that the safinamide groups also showed significant improvements in the Unified Parkinson's Disease Rating Scale (UPDRS) Part II score and the 39-item Parkinson's Disease Questionnaire (PDQ-39) score (20, 21). However, which type of patients can benefit from safinamide treatment is still largely unknown.

**Figure 1:**
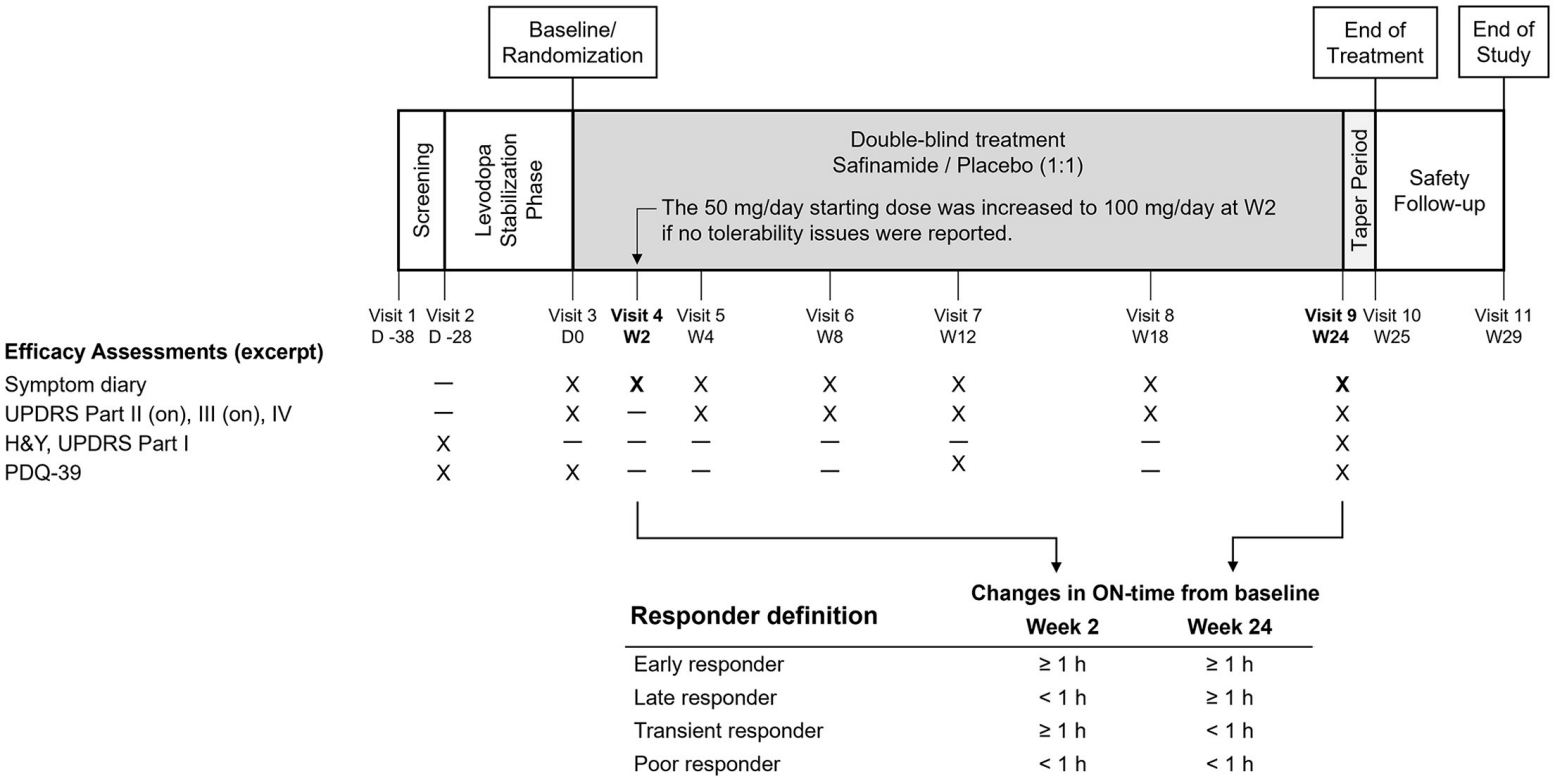
Design of the SETTLE study and the responder definition in the present analysis. H&Y, Hoehn and Yahr staging, PDQ-39, 39-item Parkinson's Disease Questionnaire; UPDRS, Unified Parkinson's Disease Rating Scale.

The present study was a *post hoc* analysis of one of the Phase III trials, the SETTLE study. The SETTLE study was a double-blind, parallel-group, 24-week trial of safinamide (50–100 mg/day orally) vs placebo in patients taking stable dosages of levodopa and concomitant PD medications (17). One of the characteristics of this study is that efficacy assessments were conducted both as early as at Week 2 and after long-term treatment for up to 6 months. By using the design of the SETTLE study, responder subgroups were defined and analyzed based on the response to treatment at Week 2 and the end of the 24-week treatment (Figure 1). The demographic characteristics and characteristics associated with treatment response were then determined in the responder subgroups.

## Methods

### Study design and study population

This was a *post hoc* analysis of a Phase III study of safinamide (SETTLE study), which was conducted in 21 countries in Europe, Asia, and North America between 2009 and 2012 and was registered at Clinical trials.gov (NCT00627640). The study was conducted in accordance with the International Conference on Harmonization Tripartite Guideline for good clinical practices and the Declaration of Helsinki. The design of the SETTLE study has been reported elsewhere (17). Briefly, the study enrolled PD patients with a disease duration of at least 3 years, Hoehn and Yahr stage 1–4, and an average daily off-time of at least 1.5 h. Before randomization, pharmacotherapy for PD was adjusted to minimize motor fluctuations and was required to be stable for at least 4 weeks. Eligible patients were then randomly assigned in a 1:1 ratio to receive safinamide or placebo. Patients received the study drug at a dose of 50 mg/day for 2 weeks, and if well-tolerated, the dose was increased to 100 mg/day (Figure 1).

### Outcome evaluations

The primary endpoint of the SETTLE study was the change from baseline to 24 weeks in ON-time without troublesome dyskinesia (hereafter, ON-time) as recorded in the 24-h diary. Other efficacy assessments included diary entries, UPDRS scores during the ON-phase, and PDQ-39 scores. For efficacy assessment at Week 2, only diary entries were evaluated (Figure 1).

### Definition of responders

The subjects were divided into four responder subgroups: Early responder, ≥ 1-h increase from baseline in ON-time both at Week 2 and at Week 24; Late responder, ≥ 1-h increase in ON-time not at Week 2, but at Week 24; Transient responder, ≥ 1-h increase in ON-time at Week 2, but not at Week 24; and Poor responder, ≥ 1-h increase in ON-time neither at Week 2 nor at Week 24 (Figure 1). The present study defined responders as subjects who achieved an increase in ON-time of at least 1 h. One reason was that this definition was in accordance with similar studies of levodopa adjuncts, in which subjects with a change in ON- or OFF-time of at least 1 h were defined as responders (22–25). Another is the reported minimal clinically important difference for OFF-time being 1.0 to 1.3 (2, 26), which supports the use of a 1-h cutoff for ON-time in the definition of responders who achieve a clinically significant change.

### Statistical methods

To analyze the responders according to the treatment responses in ON-time at Week 2 and Week 24 (end of the treatment), only those who had a diary assessment at Week 2 were included in the *post hoc* analysis population. The proportions of these responder subgroups in the safinamide and placebo groups were compared with the Chi-squared test, and the subgroup with the larger contribution to the intergroup difference was identified by analysis of residuals.

To identify factors that contributed to changes in ON-time at Week 24, multiple regression analysis was performed in each treatment group with the change in ON-time at Week 24 as the response variable and baseline values and the change in ON-time at Week 2 as explanatory variables.

To compare treatment responses by responder subgroup, summary statistics of the value at baseline and change from baseline at Week 24 were calculated by each responder subgroup and treatment group. Analysis of covariance was used to compare treatment groups within each subgroup, with the change from baseline to the Week 24 assessment as the response variable, the treatment group as the fixed effect, and the baseline value and region as the covariates.

To compare the demographic characteristics and baseline values between the early population and the others and between safinamide and placebo within subgroups, Welch's *t*-test was used for continuous variables, Fisher's exact test was used for categorical variables, and the Wilcoxon rank-sum test was used for the numbers of concomitant non-levodopa antiparkinsonian drugs.

Since this was a *post hoc* analysis, statistical methods were not pre-specified. Dropout and missing data at the last assessment point were imputed using the last observation carried forward methodology. All tests had a significance level of 5% (two-tailed), and no adjustments were made for multiplicity. All analyses were conducted using SAS version 9.4 (SAS Institute Inc., Cary, NC, USA).

## Results

### Patient population

In the SETTLE study, 274 and 275 patients were randomized to safinamide and placebo, respectively. Of these, 263 and 270 subjects, respectively, had ON-time assessment at Week 2 and were included in this *post hoc* analysis.

### Responder distribution

**Figure 2:**
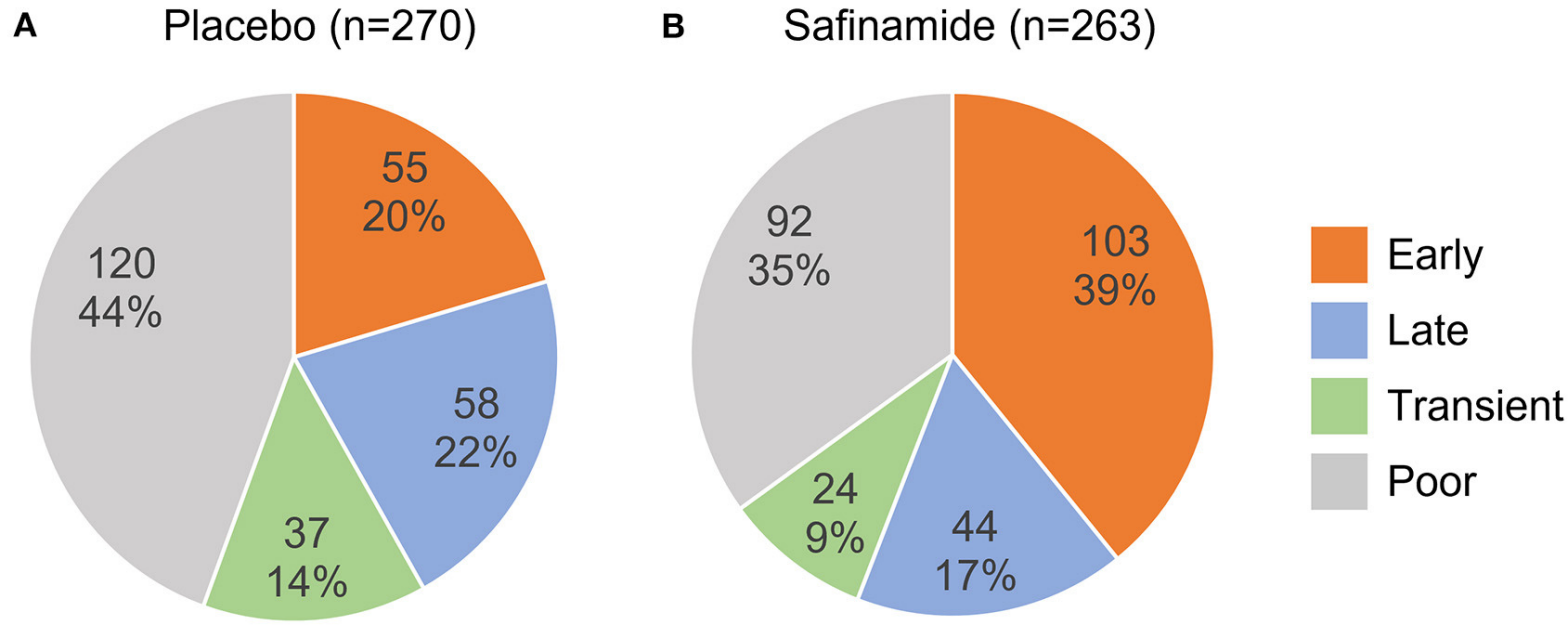
Distribution of responder subgroups in the placebo **(A)** and safinamide **(B)** groups. The upper and lower numbers are the number and the percentage, respectively, of each responder subgroup in the treatment group. The distributions are significantly different between the treatment groups (*p* < 0.0001, Chi-squared test).

The safinamide and placebo groups were divided into four responder subgroups according to the change from baseline in ON-time at Weeks 2 and 24 of treatment (Figure 1). In the safinamide group, more than one-third of the subjects were classified as Early responders (103 subjects, 39%), another one-third were Poor responders (92, 35%), and the Late and Transient responders were few (44, 17; 24, 9%, respectively) (Figure 2). On the other hand, in the placebo group, the largest population was the Poor responders (120, 44%), followed by the Late responders (58, 22%), the Early responders (55, 20%), and the Transient responders (37, 14%). The distribution of the responders was significantly different between the safinamide and placebo groups (*p* < 0.0001, Chi-squared test), with significant differences in the proportion of Early responders (*p* < 0.0001) and Poor responders (*p* = 0.03).

The proportion of responders who achieved an increase in ON-time of at least 1 h as of Week 24, i.e., the total of Early and Late responders, was significantly higher (56%) in the safinamide group than in the placebo group (42%; *p* = 0.001, Chi-squared test).

In the safinamide group, 127 subjects showed a 1-h or greater increase in ON-time at Week 2, and most of them (103 subjects, 81%) maintained the treatment response at Week 24. A similar trend was found in the placebo group: 55 of 92 (60%) subjects who showed a response at Week 2 maintained their response at Week 24, but the rate was significantly greater in the safinamide group than in the placebo group (*p* = 0.0005, Chi-squared test).

These results were supported by the multivariable regression analyses, which showed that the change in ON-time from baseline to Week 2 was positively associated with the change in ON-time from baseline to Week 24 in both treatment groups (Supplementary Table 1).

### Treatment responses by responder subgroups

**Figure 3:**
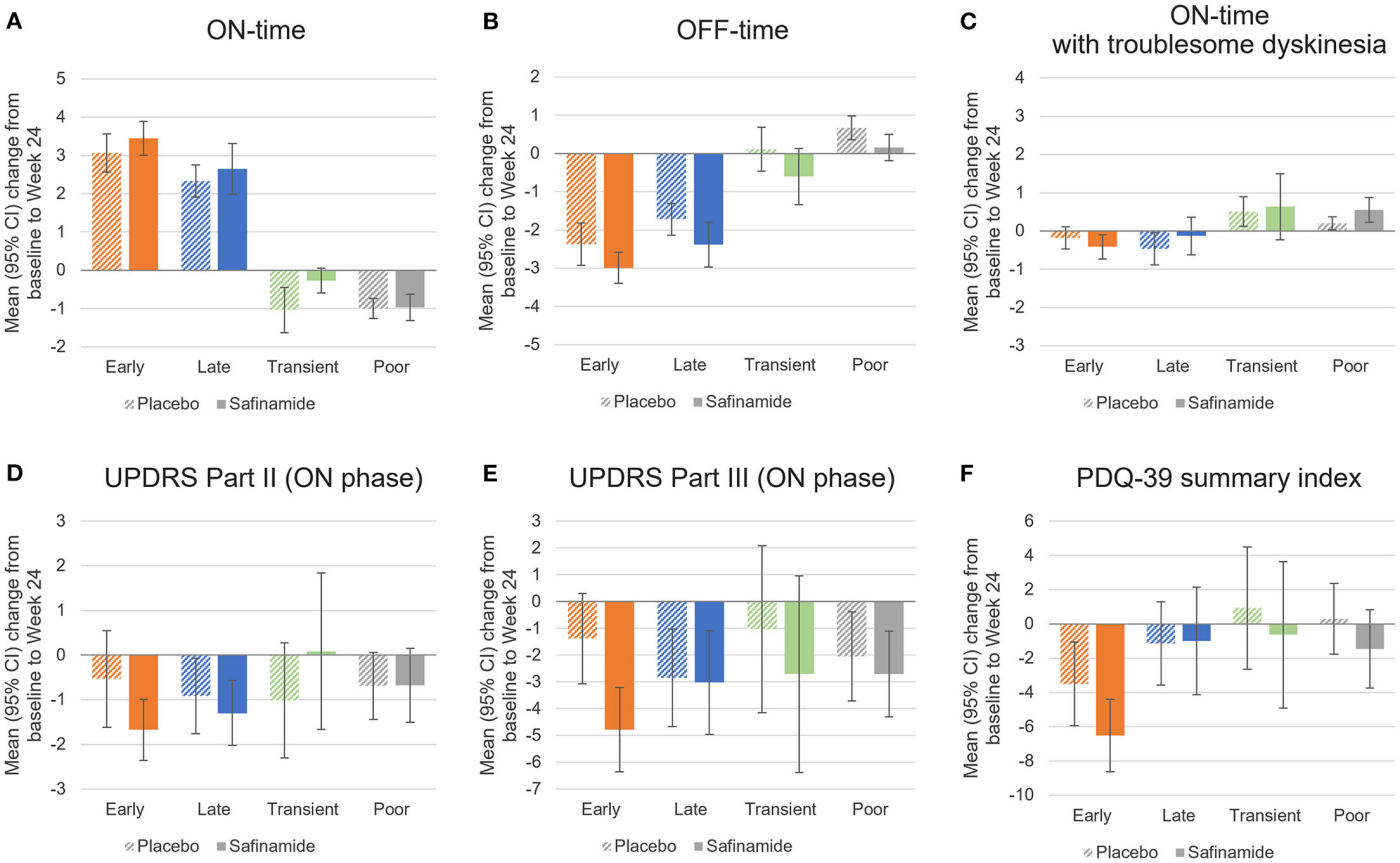
Changes from baseline to Week 24 in diary outcomes **(A–C)**, UPDRS scores **(D, E)**, and the PDQ-39 summary index **(F)** by responder subgroup in the placebo and safinamide groups. 95%CI, 95% confidence interval; PDQ-39, 39-item Parkinson's Disease Questionnaire; UPDRS, Unified Parkinson's Disease Rating Scale.

Changes in the outcomes from baseline at Week 24 in the individual responder subgroups are shown in Figure 3, Supplementary Figure 1, and Supplementary Tables 2–4. Reflective of the defining of responders according to changes in ON-time, ON-time changes in the responder subgroups did not differ substantially between the safinamide and placebo groups. Changes in scores in UPDRS Parts II and III and the PDQ-39 summary index, however, did not necessarily mirror this trend in ON-time changes: UPDRS Part III scores, for example, decreased substantially from baseline even among the Poor responders.

At Week 24, the Early responders in the safinamide group achieved a substantial reduction from baseline in OFF-time, UPDRS Part II and III scores, and PDQ-39 summary index scores. The change in the UPDRS Part III score was significant compared with the Early responders in the placebo group. At Week 24, the Late responders achieved a reduction from baseline in OFF-time and UPDRS Part II and III scores. Their reduction in OFF-time was significant relative to the placebo group. The few Transient responders had broad confidence intervals in changes in UPDRS and PDQ-39 scores. The Poor responders in the safinamide group had a significant reduction in OFF-time and a significant increase in ON-time with troublesome dyskinesia compared with the placebo group.

### Baseline characteristics

The demographic characteristics, baseline values, and concomitant use of antiparkinsonian drugs in the different responder subgroups are summarized in Table 1 and Supplementary Table 5. In the safinamide group, the Early responders had, at baseline, significantly higher UPDRS Part II and III scores, shorter ON-time, and longer OFF-time than the other responder populations (Table 1). In the placebo group as well, the Early responders had significantly shorter ON-time and longer OFF-time than the other responder populations, but UPDRS Part II and III scores did not differ significantly and tended to be lower in the Early responders (Supplementary Table 5). No significant differences were seen in the other demographic characteristics, baseline values, or concomitant drugs. A comparison of the safinamide and placebo groups in each responder subgroup showed significantly higher UPDRS Part II and III and total scores in the safinamide group than in the placebo group among Early responders (35.78 vs. 29.57, *p* = 0.04) and significantly lower UPDRS Part II and III and total scores among the Poor responders (30.36 vs. 34.88, *p* = 0.04).

**Table 1:** Demographic and baseline characteristics by responder subgroup in the safinamide group.

			**Early**	**Late**	**Transient**	**Poor**	***p*-value (early vs. others)**
			**(*****N*** = **103)**	**(*****N*** = **44)**	**(*****N*** = **24)**	**(*****N*** = **92)**	
Sex, male		*n* (%)	63 (61.2)	23 (52.3)	13 (54.2)	65 (70.7)	0.7948
Age, years		Mean (SD)	62.13 (8.91)	61.61 (8.75)	62.29 (8.72)	61.29 (9.48)	0.6009
Weight, kg		Mean (SD)	74.76 (16.57)	69.45 (17.85)	72.98 (13.93)	70.83 (16.37)	0.0585
Duration of PD, years		Mean (SD)	8.97 (4.75)	9.03 (4.46)	8.91 (4.01)	8.68 (4.12)	0.7783
Young onset PD (<50 years old)		*n* (%)	37 (35.9)	17 (38.6)	8 (33.3)	34 (37.0)	0.8963
H&Y stage		Mean (SD)	2.47 (0.62)	2.53 (0.54)	2.46 (0.51)	2.51 (0.58)	0.5947
UPDRS Part I		Mean (SD)	1.26 (1.15)	1.43 (1.32)	1.00 (1.29)	1.32 (1.57)	0.8149
UPDRS Part II (ON phase)		Mean (SD)	11.13 (5.21)	9.84 (5.59)	8.50 (6.14)	9.33 (5.49)	0.0096
UPDRS Part III (ON phase)		Mean (SD)	24.91 (13.57)	21.64 (10.58)	20.21 (8.85)	21.08 (9.71)	0.0146
UPDRS Part IV		Mean (SD)	6.30 (2.97)	5.55 (2.78)	6.17 (3.46)	5.66 (2.84)	0.1108
Daily ON-time without troublesome dyskinesia, hours		Mean (SD)	8.24 (2.49)	9.26 (2.15)	9.25 (2.19)	10.44 (2.03)	<0.0001
Daily ON-time with troublesome dyskinesia, hours		Mean (SD)	1.16 (2.13)	1.06 (2.07)	0.97 (2.17)	0.55 (1.19)	0.0983
Daily OFF-time, hours		Mean (SD)	5.93 (2.03)	5.31 (2.01)	5.89 (1.90)	4.59 (1.68)	0.0002
PDQ-39 summary index score		Mean (SD)	29.07 (14.22)	27.03 (13.96)	26.52 (13.54)	26.78 (15.93)	0.2190
Levodopa dose, mg/day		Mean (SD)	790.58 (482.79)	701.42 (291.90)	724.58 (520.57)	780.93 (463.77)	0.4940
Levodopa equivarent dose, mg/day		Mean (SD)	1,048.01 (453.78)	1,025.56 (417.79)	934.51 (425.84)	988.44 (492.83)	0.3257
Concomitant use of non-levodopa antiparkinsonian drugs		*n* (%)	91 (88.3)	38 (86.4)	23 (95.8)	87 (94.6)	0.2777
Dopamine agonists		*n* (%)	75 (72.8)	30 (68.2)	21 (87.5)	71 (77.2)	0.5617
Entacapone		*n* (%)	20 (19.4)	6 (13.6)	5 (20.8)	15 (16.3)	0.5111
Carbidopa/levodopa/entacapone		*n* (%)	36 (35.0)	15 (34.1)	7 (29.2)	22 (23.9)	0.2181
Amantadine		*n* (%)	31 (30.1)	21 (47.7)	5 (20.8)	25 (27.2)	0.7866
Anticholinergic agents		*n* (%)	13 (12.6)	9 (20.5)	2 (8.3)	21 (22.8)	0.1338
Number of concomitant non levodopa antiparkinsonian drugs	0	*n* (%)	12 (11.7)	6 (13.6)	1 (4.2)	5 (5.4)	0.9254
	1	*n* (%)	31 (30.1)	10 (22.7)	9 (37.5)	36 (39.1)	
	2	*n* (%)	39 (37.9)	16 (36.4)	12 (50.0)	35 (38.0)	
	3	*n* (%)	18 (17.5)	9 (20.5)	1 (4.2)	16 (17.4)	
	4	*n* (%)	3 (2.9)	3 (6.8)	1 (4.2)	0 (0.0)	

H&Y, Hoehn and Yahr; PD, Parkinson's disease; PDQ-39, 39-item Parkinson's Disease Questionnaire; SD, standard deviation; UPDRS, Unified Parkinson's Disease Rating Scale.

## Discussion

The present study demonstrated that the safinamide group in the SETTLE study, relative to the placebo group, had a significantly higher proportion of Early responders, i.e., subjects who achieved a clinically significant increase in ON-time early in treatment and maintained the response through Week 24. The change in Part III scores was significantly greater in the Early responders in the safinamide group than in the placebo group. Although the Early responders in both treatment groups had substantial improvements from baseline in OFF-time, UPDRS Part II and III scores, and PDQ-39 summary index scores through Week 24, the safinamide group had greater changes in Part II and III and PDQ-39 summary index scores than the placebo group. The differences were not significant; however, they exceeded the minimal clinically important differences (Part II, −0.7; Part III, −2.4; PDQ-39 summary index, −1.6) (2, 3). This finding suggests that Early responders are one group of patients that benefits greatly from safinamide treatment.

Characteristically, Early responders achieve a large early improvement in ON-time at Week 2 that persists through Week 24. In the safinamide group, about 80% of the subjects with an improvement of at least 1 h in ON-time at Week 2 were Early responders. Change from baseline in ON-time at Week 2 was significantly associated with the change at Week 24. Thus, the initial response may help to predict the long-term therapeutic response to safinamide.

The baseline shorter ON-time was another characteristic of Early responders. Reportedly, the more severe the symptoms at baseline were, the larger the magnitude of therapeutic response would be expected (10, 11, 27). For example, Poewe et al. showed that baseline OFF-time was significantly correlated with levodopa/carbidopa intestinal gel-induced OFF-time improvement at month 24 (27). This relationship was also found in the placebo-treated subjects (28). Patients with shorter ON-time would have a higher expectation for the study medication to elongate ON-time, so they might show greater response in ON-time.

Early responders in the safinamide group also had higher UPDRS Part II and III scores at baseline, and importantly, this was not found in the placebo group. These baseline characteristics indicated that Early responders in the safinamide group were those whose baseline treatment was suboptimal for symptomatic improvement during the ON phase. Since Early responders showed a tendency toward a higher UPDRS Part IV score and longer ON-time with troublesome dyskinesia at baseline, dyskinesia during the ON-phase might limit dopaminergic treatments in this population. Nonetheless, safinamide treatment brought a large increase in ON-time without troublesome dyskinesia, with a slight decrease in ON-time with troublesome dyskinesia from baseline in Early responders. This is in accordance with the previous reports, which showed that long-term safinamide treatment did not exacerbate dyskinesia in a subgroup of patients with dyskinesia at baseline (29, 30). Safinamide has both dopaminergic and non-dopaminergic actions, and this “dual action” may help overcome the limit of dopaminergic treatments in Early responders. For example, safinamide was reported to inhibit glutamate release in the basal ganglia in rats *via* its sodium channel inhibitory effect (15). Excessive glutamate signaling has been suggested in dyskinesia pathophysiology (31, 32), and abnormal cortical facilitation, which indicates hyperactive glutamatergic neurotransmission, was reported in patients with levodopa-induced dyskinesia (33). Guerra et al. have shown that long-term safinamide treatment ameliorated cortical facilitation, and the change in cortical facilitation and the change in dyskinesia severity were positively correlated (34). Although it is not clear that the non-dopaminergic action of safinamide was involved in the decrease in cortical facilitation, this suggests that inhibition of glutamatergic neurotransmission may lead to dyskinesia alleviation.

The proportions of patients on placebo and safinamide in the different responder subgroups differed significantly. The proportion of Early responders in the safinamide group (39%) was almost double that in the placebo group (20%), which suggests that safinamide is effective in reducing wearing-off. Likewise, the proportion of subjects whose ON-time was increased at least 1 h at Week 24 (i.e., Early + Late responders) was significantly higher in the safinamide group (56%) than in the placebo group (42%). This high responder rate at Week 24 was reproduced in a Japanese Phase II/III study of safinamide (18). In addition, it is comparable to the responder rates found in the clinical studies of other levodopa adjuncts for advanced PD (22–25). For example, in the LARGO study, the proportions of subjects with a decrease from baseline of at least 1 h in OFF-time at Week 18 were 51% in the rasagiline 1 mg group, 45% in the entacapone 200 mg group, and 32% in the placebo group (22). In a Phase III clinical study of opicapone, the proportions of subjects with an increase in ON-time of at least 1 h at Week 14–15 was 57% in the opicapone 25 mg group, 65% in the opicapone 50 mg group, 58% in the entacapone 200 mg group, and 46% in the placebo group (25).

The proportion of Transient responders in the safinamide group (24 of 263 subjects, or 9%) was not substantially different from that in the placebo group (37 of 270 subjects, or 14%). Since beginning treatment often entails a psychological placebo effect, the transient responses seen may be indicative of such an effect. Alternatively, Transient responders may have rapid disease progression that may mask long-term efficacy. It is also possible to assume that the Transient responders may have responded only to 50 mg because the subjects in the SETTLE study started safinamide treatment at a dose of 50 mg/day, and about 90% of them were subsequently escalated to 100 mg/day at Week 2 (17). A lack of response to high doses was found in a clinical study in patients with early PD (Study 015) (35): Although treatment with 100 mg of safinamide in Study 015 significantly improved UPDRS Part III scores over placebo, no significant improvement was achieved at the higher dose of 200 mg. The present analysis may have shown the presence of a population of transient responders among PD patients with wearing-off who respond sufficiently to 50 mg of safinamide and gain no benefits from higher doses. If this proves true, proper dose adjustment should help maintain a therapeutic effect. The reason for the lack of efficacy at the high dose in Study 015, however, is unclear (35). A high dropout rate in the higher dose group was also proposed as a cause of the lack of efficacy. It is, therefore, unknown whether a lack of efficacy at higher doses could be seen in clinical practice.

The outcomes at Week 24 were more varied among the subjects without an improvement of at least 1 h in ON-time at Week 2. One-third of them (Late responders) achieved an increase of more than 1 h in ON-time at Week 24, and the rest did not (Poor responders). Furthermore, even in the Poor responders, some outcomes other than ON-time were improved; for example, the safinamide group had a significantly greater decrease in OFF-time from baseline through Week 24 than the placebo group, and the difference between the safinamide and placebo groups in the change in PDQ-39 summary index scores exceeded the minimal clinically important difference (3). Given the nature of PD with its diverse symptoms, the continuation of treatment should be considered with their problematic symptoms factored in.

The present study has several limitations. First, it was a *post hoc* analysis with no predefined conditions. Given that the responder subgroups were defined by post-baseline characteristics, the present study did not adequately control the demographic characteristics and baseline values of the responders among the groups. Second, since subjects in the SETTLE study who tolerated 50 mg/day were escalated to 100 mg/day (17), it is unclear whether the initial treatment response is predictive of the long-term response when the dose is maintained at 50 mg/day. Moreover, as stated previously, the dose may not have been optimized in some patients. Third, the present study defined responders as subjects who achieved an increase in ON-time of at least 1 h. Some studies, however, have defined responders as those subjects who achieved a change from baseline in ON- or OFF-time of at least 30% or at least 3 h (11, 27, 36–38). The use of different cutoff values may yield different results.

In conclusion, the present *post hoc* analysis showed that, when a patient responded significantly to safinamide treatment early on, the response was more likely to last for a longer period of time. Furthermore, patients with a short ON-time, severe motor symptoms, and highly compromised activities of daily living may benefit from safinamide early in the treatment and over the long term. These results suggest that the evaluation of activities of daily living, as well as wearing-off and motor functions, is crucial for the most effective treatment with safinamide. Recently, genetic biomarkers for other antiparkinsonian drugs that predict patients' therapeutic responses have been discovered (12–14). To provide precision treatment with safinamide, further research may be warranted to discover biomarkers that predict response to safinamide treatment.

## Data availability statement

The data analyzed in this study was obtained from Eisai Co., Ltd, the following licenses/restrictions apply: access to these datasets must be approved by Eisai Co., Ltd. Requests to access these datasets should be directed to RB, rbh@chulapd.org.

## Ethics statement

The studies involving human participants were reviewed and approved by an appropriately constructed Independent Ethics Committee. The patients/participants provided their written informed consent to participate in this study.

## Author contributions

Statistical analysis: IS. Preparation of the first draft: MK. Conception and design of the study, interpretation of data, review and critique of the statistical analysis, and the entire draft: All authors. All authors have approved the final manuscript.

## Supplementary material

Supplementary Figure 1 Change from baseline in ON-time by responder subgroup.

Supplementary Table 1 Multivariable regression analysis to find factor(s) associated with change from baseline to Week 24 in ON-time.

Supplementary Table 2 The baseline values and changes from baseline to Week 24 in diary outcomes by responder subgroups in the placebo and safinamide groups.

Supplementary Table 3 The baseline values and changes from baseline to Week 24 in UPDRS Part II and III scores by responder subgroups in the placebo and safinamide groups.

Supplementary Table 4 The baseline values and changes from baseline to Week 24 in PDQ-39 summary index and subdomain scores by responder subgroups in the placebo and safinamide groups.

Supplementary Table 5 Demography and baseline characteristics by responder subgroups in the placebo group.
