# Supplementary figures and images for: Sustained response in early responders to safinamide in patients with Parkinson's disease and motor fluctuations: A post hoc analysis of the SETTLE study
Source: Front Neurol. 2023 Mar 27;14:1147008. doi: 10.3389/fneur.2023.1147008 (PMC10083404; doi:10.3389/fneur.2023.1147008)

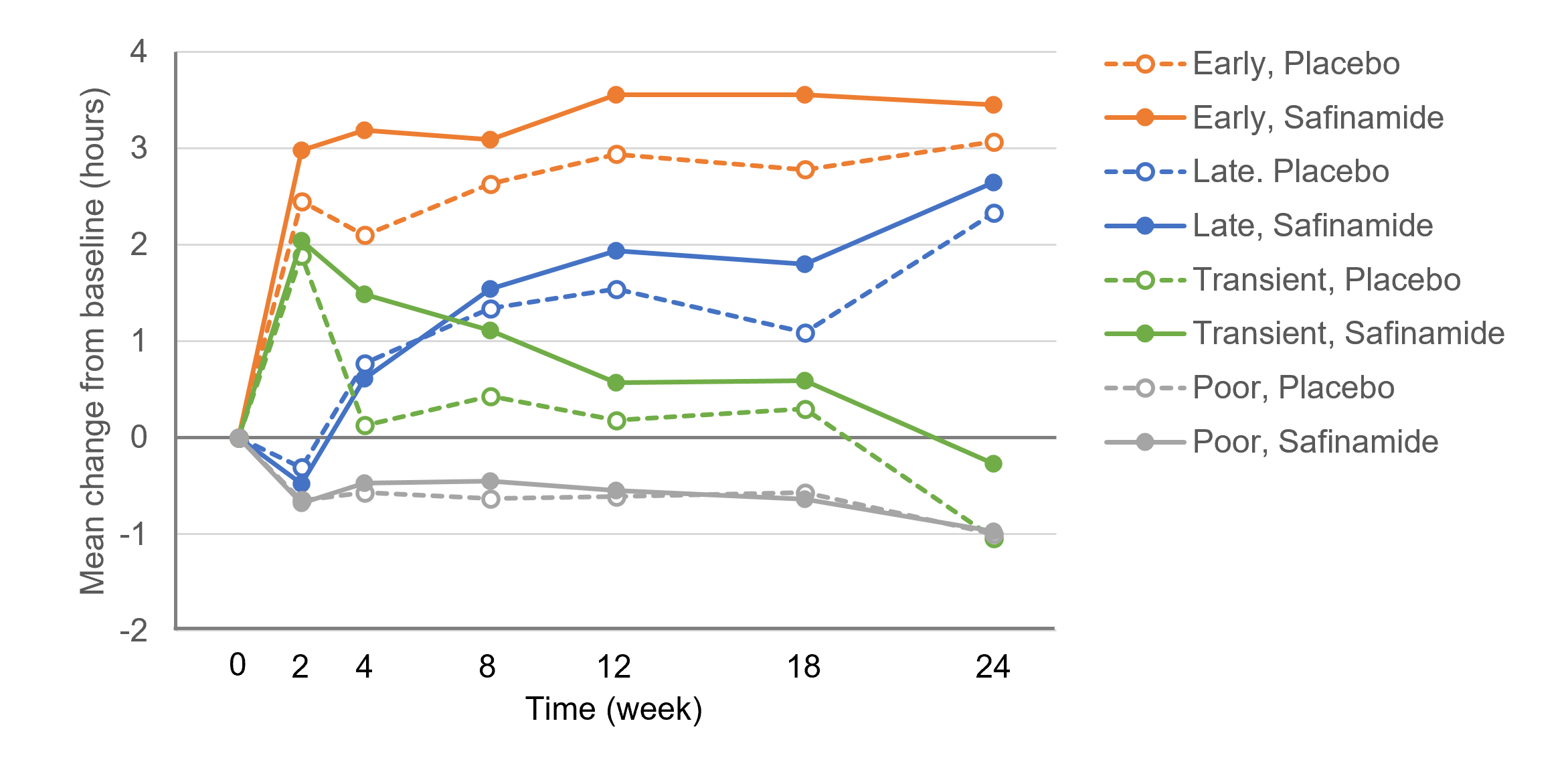

Supplement: Supplementary Figure 1 — Change from baseline in ON-time by responder subgroup. [file Image_1.TIF]
